# Transcriptomic analysis identifies FABP5 as a candidate regulator of lipid accumulation in metabolic dysfunction-associated fatty liver disease

**DOI:** 10.3389/fendo.2026.1883460

**Published:** 2026-08-31

**Authors:** Kaishuo Lv, Shuhang Zhong, Xiaoqing Liu, Junru Zhou, Di Lu, Xiaoying Luo, Liqun Tu, Bingxi Zhou, Shuangyin Han, Bingyong Zhang, Xiuling Li, Jiayu (Daisy) Ye, Zhiyu Yang

**Affiliations:** 1 Department of Gastroenterology and Hepatology,Henan Provincial People’s Hospital, People's Hospital of Zhengzhou University, Zhengzhou, China; 2 Department of Gastroenterology and Hepatology, Henan Provincial People’s Hospital, People's Hospital of Henan University, Zhengzhou, China; 3 Department of Laboratory Medicine, Henan Provincial People's Hospital, People's Hospital of Zhengzhou University, Zhengzhou, China; 4 Department of Gastroenterology and Hepatology, Stanford University School of Medicine, Palo Alto, CA, United States

**Keywords:** fatty acid-binding protein 5, lipid transport and metabolism, metabolic dysfunction-associated fatty liver disease, therapeutic target, transcriptome analysis

## Abstract

**Objective:**

The pathogenesis of metabolic dysfunction-associated fatty liver disease (MAFLD) is very complex, which has not been fully revealed as so far. This study aimed to identify differentially expressed genes involved in the pathogenesis of MAFLD using Oxford Nanopore Technologies (ONT) transcriptomic sequencing and to investigate the potential role of fatty acid-binding protein 5 (FABP5) in hepatic lipid accumulation.

**Methods:**

Male C57/BL6N mice with fed either a high-fat, high-fructose (HFHF) diet for 19 weeks to induce MAFLD or a standard chow diet. Liver tissues were subjected to histological examination and ONT transcriptomic analysis and selected differentially expressed genes involved in lipid transport and metabolism were validated by quantitative reverse-transcription PCR (RT-qPCR). Serum FABP5 concentrations were measured in HFHF-fed mice and patients with MAFLD using enzyme-linked immunosorbent assay (ELISA). *In vitro*, Huh-7 cells were treated with different concentrations of palmitic acid (PA) to induce lipid accumulation. FABP5 was subsequently overexpressed in PA-treated cells, followed by assessment of intracellular lipid accumulation and the expression of genes involved in fatty acid oxidation.

**Result:**

HFHF-fed mice exhibited marked hepatic steatosis, hepatocyte ballooning, and lobular inflammation. ONT transcriptomic analysis identified 400 differentially expressed genes between the HFHF and chow groups, including 12 genes involved in lipid transport and metabolism. RT-qPCR validation showed that hepatic *Fabp5* expression was significantly decreased in HFHF-fed mice, consistent with the ONT sequencing results. Serum FABP5 concentrations were significantly lower in HFHF-fed mice and patients with MAFLD than in their respective controls. PA treatment induced intracellular lipid accumulation in Huh-7 cells in a concentration-dependent manner. FABP5 overexpression attenuated PA-induced lipid accumulation and increased *PPARα* expression, together with changes in other genes associated with fatty acid oxidation.

**Conclusion:**

Hepatic *Fabp5* expression and circulating FABP5 concentrations are reduced in experimental and human MAFLD. Restoration of FABP5 attenuates PA-induced lipid accumulation in hepatocyte-derived cells and is accompanied by increased expression of *PPARα* and other fatty acid oxidation-related genes. These findings suggest that FABP5 is a candidate regulator of hepatic lipid metabolism and warrants further investigation as a potential therapeutic target for MAFLD. Additional inhibition and rescue experiments are required to determine whether PPARα is a necessary mediator of the lipid-lowering effect of FABP5.

## Introduction

Nonalcoholic fatty liver disease (NAFLD) is characterized by excessive hepatic lipid accumulation in the absence of substantial alcohol consumption or other identifiable causes of liver injury. Its clinical spectrum ranges from simple steatosis to nonalcoholic steatohepatitis, fibrosis, cirrhosis, and hepatocellular carcinoma, and it affects approximately one-quarter of the global population (1–4). Recognition of the central role of systemic metabolic dysfunction in fatty liver disease led to the proposal of the term metabolic dysfunction-associated fatty liver disease (MAFLD), which emphasizes the coexistence of hepatic steatosis with overweight or obesity, type 2 diabetes, or other metabolic abnormalities (5–10).

Dysregulated hepatic lipid metabolism is a major feature of MAFLD. Increased fatty acid uptake and triglyceride synthesis together with impaired fatty acid oxidation and lipid export, contribute to hepatic steatosis and disease progression (11–14). Transcriptomic studies have identified alterations in genes involved in fatty acid transport, lipid droplet formation, triglyceride synthesis, and fatty acid oxidation, providing an approach for identifying candidate regulators of MAFLD (15). Fatty acid-binding protein 5 (FABP5) is a 14–15-kDa cytosolic lipid chaperone that binds long-chain fatty acids and regulates their intracellular transport, metabolic utilization, and delivery to nuclear receptors (16–22). FABP5 has been linked particularly to PPARβ/δ-associated lipid metabolic programs and has been implicated in systemic energy homeostasis and inflammatory responses (23, 24). However, its expression and function in fatty liver disease appear to vary according to disease context, tissue compartment, and cellular source, and evidence regarding circulating FABP5 in MAFLD remains limited (25–29).

In the present study, we used Oxford Nanopore Technologies transcriptomic sequencing to characterize hepatic gene-expression changes in mice with HFHF diet-induced MAFLD and identified *Fabp5* as a differentially expressed gene involved in lipid transport and metabolism. We subsequently evaluated hepatic *Fabp5* expression and circulating FABP5 concentrations in experimental and human MAFLD. Finally, we investigated whether FABP5 overexpression affected PA-induced lipid accumulation and the expression of fatty acid oxidation-related genes in Huh-7 cells. This study aimed to clarify the association of FABP5 with MAFLD and explore its potential role in hepatocellular lipid metabolism.

## Materials and methods

### Animals and treatment

Male C57BL/6N mice (6–8 week-old) were purchased from Beijing Vital River Laboratory Animal Technology Co. Ltd (Beijing, China). The mice were housed under controlled conditions at a constant temperature of 25 ± 2 °C with a 12-h light/12-h dark cycle and were provided free access to food and water. All animal experimental procedures were approved by the Animal Ethics Committee of Zhengzhou University.

For the high-fat, high-fructose (HFHF) diet-induced MAFLD model, mice were randomly assigned to either the control group or the HFHF group (n=4 per group). The control group was fed a Stanford chow diet, whereas the HFHF group received a diet containing 60% fat together with fructose-supplemented drinking water at a concentration of 42 g/L for 19 weeks. Both diet were obtained from Jiangsu Medicience Biomedical Co.Ltd (Yangzhou, China). At the end of the feeding period, the mice were sacrificed, and serum and liver tissue samples were collected for subsequent analyses.

For the methionine- and choline-deficient (MCD) diet-induced model, a separate cohort of mice was randomly divided into a chow-fed control group and two MCD-fed experimental groups (n=5 per group). Mice in the experimental groups were fed an MCD diet for either 2 or 8 weeks, whereas the corresponding control mice continued to receive the standard chow diet for 8 weeks. The MCD diet was also obtained from Jiangsu Medicience Biomedical Co. Ltd (Yangzhou, China). At the indicated time points, the mice were sacrificed, and serum and liver tissues were collected for subsequent analyses.

### Histological analysis

Liver tissues were fixed in paraformaldehyde, embedded in paraffin, sectioned, and stained with Hematoxylin and Eosin (H&E) or Sirius Red. Hepatic steatosis, lobular inflammation, and hepatocellular ballooning were evaluated using the NAFLD Activity Score (NAS) system established by Kleiner et al. (30). Steatosis was scored according to the proportion of hepatocytes containing lipid droplets as follows: 0, <5%; 1, 5–33%; 2, >33–66%; and 3, >66%. Hepatocellular ballooning was scored as follows: 0, none; 1, few balloon hepatocytes; and 2, many balloon hepatocytes or prominent ballooning. Lobular inflammation was scored according to the number of inflammatory foci per ×200 microscopic field as follows: 0, no foci; 1, <2 foci; 2, 2–4 foci; and 3, >4 foci. The NAS was calculated as the sum of the steatosis, lobular inflammation, and hepatocellular ballooning scores, yielding a total score ranging from 0 to 8. Hepatic collagen deposition was quantified in Sirius Red-stained sections as the percentage of positively stained area using ImageJ software (National Institutes of Health, USA). Histological evaluation was performed independently by two investigators who were blinded to the experimental groups, and discrepancies were resolved by consensus.

### Biochemical analysis

Blood samples were allowed to clot at room temperature for 30 minutes and then centrifuged at 2,000 × g for 10 min at 4 °C to separate the serum. The resulting serum was collected, aliquoted, and stored at −80 °C until analysis. Serum alanine aminotransferase (ALT) and aspartate aminotransferase (AST) activities were measured as biochemical indices of hepatocellular injury, while serum total cholesterol (TC) and triglyceride (TG) concentrations were measured to assess circulating lipid abnormalities. All biochemical measurements were performed using a Hitachi 7180 automated biochemical analyzer (Hitachi High-Tech Corporation, Japan) according to the manufacturer’s instructions. ALT and AST activities are reported in U/L, whereas TC and TG concentrations are reported in mmol/L.

### Gene-expression profiling and bioinformatic analysis

Oxford Nanopore Technologies (ONT) sequencing (61) and the associated bioinformatic analyses were performed by Biomarker Technologies Corporation (Beijing, China). The analytical workflow is summarized in **Supplementary Figure 1**. RNA purity, concentration, and integrity were assessed before library preparation. For each sample, 1 μg of total RNA was used to construct a full-length cDNA library using the ONT cDNA-PCR Sequencing Kit (SQK-PCS109) according to the manufacturer’s protocol. Template-switching reverse transcription was used to enrich full-length cDNA molecules and introduce defined PCR adapters at both ends of the first-strand cDNA. The resulting cDNA was amplified for 14 PCR cycles using LongAmp Taq DNA polymerase (New England Biolabs, USA). Sequencing adapters were ligated using T4 DNA ligase (New England Biolabs, USA), and the products were purified using Agencourt AMPure XP beads. The final libraries were loaded onto FLO-MIN109 flow cells and sequenced on the PromethION platform.

Raw electrical-signal data in FAST5 format were base-called and converted to FASTQ format using Guppy implemented within MinKNOW (version 2.2). Reads with an average quality score <7 or a length <500 bp were discarded and reads assigned to ribosomal RNA were subsequently removed. Across the eight libraries, 24,283,325 clean reads, corresponding to approximately 30.54 Gb of sequence data, passed the quality- and length-based filters. After removal of 5,313,250 rRNA-derived reads, 18,970,075 non-rRNA reads remained. Primer sequences at both ends of each read were identified to classify full-length non-chimeric reads, yielding 16,262,565 such reads across the eight samples.

Full-length non-chimeric reads were aligned to the mouse GRCm38 reference genome using minimap2 and clustered according to their genomic alignments. Transcript clusters were polished using the Pinfish pipeline to generate consensus isoforms. The consensus sequences were realigned to GRCm38 using minimap2 and collapsed using cDNA_Cupcake with minimum alignment-coverage and sequence-identity thresholds of 85% and 90%, respectively. Differences confined to the 5′ ends were ignored when collapsing redundant transcripts. This procedure generated 18,238 non-redundant transcript sequences.

For expression quantification, full-length reads were mapped to the reference transcriptome and reads with a mapping quality score (MAPQ) >5 were retained. Transcript-level counts assigned to the same gene were summed to generate a gene-level raw read-count matrix. A total of 15,372 genes had at least one mapped read in one or more of the eight samples and were considered detected. CPM values were calculated separately to describe and visualize relative expression levels but were not used as the statistical input for differential-expression testing.

Differential-expression analysis was performed using the gene-level raw read-count matrix and DESeq2 (R package, version 1.6.3). The analysis included four chow-fed and four HFHF-fed mice and used dietary group as the sole explanatory variable (design = ~ diet_group), with the chow group as the reference level. No additional covariates or batch terms were included in the model. DESeq2 estimated sample-specific size factors to account for differences in sequencing depth and library composition and modeled gene-wise read counts using a negative binomial distribution. Differences between the HFHF and chow groups were assessed using the Wald test. The resulting *P* values were adjusted using the Benjamini–Hochberg procedure to control the false discovery rate. Genes with |log_2_FC| ≥1, corresponding to an absolute fold change ≥2, and an FDR <0.01 were defined as differentially expressed genes (DEGs).

Protein-coding DEGs were functionally annotated using the Gene Ontology (GO), Kyoto Encyclopedia of Genes and Genomes (KEGG), and eggNOG databases. GO enrichment analysis was performed using the GOseq R package with the Wallenius non-central hypergeometric distribution to account for gene-length bias, whereas KEGG pathway enrichment analysis was performed using KOBAS. To identify genes involved in lipid metabolism, the protein-coding DEG list was intersected with genes assigned to eggNOG functional category I, “lipid transport and metabolism.” This predefined annotation-based filtering procedure identified 12 lipid transport- and metabolism-related DEGs, all of which were subjected to subsequent RT-qPCR validation.

### Cell culture

The human hepatocellular carcinoma-derived cell line Huh-7 was cultured in Dulbecco’s modified Eagle’s medium (DMEM; Corning, USA) supplemented with 10% fetal bovine serum (FBS; Thermo Fisher Scientific, USA) and 1% penicillin-streptomycin (Beyotime Biotechnology, China). Cells were maintained at 37 °C in a humidified incubator containing 5% CO_2_.

### Plasmid transfection

The FABP5 expression plasmid and corresponding empty-vector plasmid were purchased from Sangon Biotech (Shanghai, China). Huh-7 cells were transfected using Lipofectamine 2000 reagent (Thermo Fisher Scientific, USA) according to the manufacturer’s instructions. For each well, 10 μL of Lipofectamine 2000 was diluted in 250 μL of Opti-MEM (Thermo Fisher Scientific, USA) and incubated at room temperature for 5 min. In parallel, 4 μg of plasmid DNA was diluted in 250 μL of Opti-MEM. The diluted Lipofectamine 2000 and plasmid DNA were combined, mixed gently, and incubated at room temperature for 30 min to allow formation of DNA–lipid complexes. The resulting 500 μL transfection mixture was added to each well. After incubation for 4–6 h at 37 °C in 5% CO_2_, the transfection medium was replaced with complete growth medium. Cells were subsequently cultured for 24–48 h before downstream experiments. Cells transfected with the FABP5 expression plasmid were designated the FABP5-overexpression group, while untransfected cells and cells transfected with the empty vector served as the control and vector groups, respectively.

### Palmitic acid treatment and oil red O staining

A PA-induced lipid-accumulation model was established in Huh-7 cells. PA powder was dissolved in 0.1 M NaOH at 70 °C to prepare a 100 mM PA solution. The solution was subsequently combined with 10% fatty acid-free bovine serum albumin (BSA; Thermo Fisher Scientific, USA) and incubated at 55 °C for 1 h to allow PA–BSA conjugation, yielding a 10 mM PA stock solution. The PA–BSA stock solution was passed through a 0.22-μm filter and stored at −20 °C until use. Before treatment, the stock solution was diluted in complete culture medium to the indicated final concentrations. Huh-7 cells were treated with 0.4, 0.6, or 0.8 mmol/L PA for 24 h to evaluate concentration-dependent lipid accumulation. For the FABP5-overexpression experiments, transfected cells were treated with 0.6 mmol/L PA for 24 h.

Intracellular lipid accumulation was evaluated using an Oil Red O staining kit (Solarbio, Beijing, China) according to the manufacturer’s instructions. Stained cells were imaged under a light microscope, and Oil Red O staining was quantified using ImageJ software (National Institutes of Health, Bethesda, MD, USA).

### Quantitative reverse-transcription PCR analysis

Quantitative reverse-transcription PCR (RT-qPCR) was performed to validate the ONT transcriptomic results and to measure gene expression in Huh-7 cells. Total RNA was extracted from mouse liver tissues or cultured cells using TRIzol™ Reagent (Invitrogen, Thermo Fisher Scientific, USA). RNA was reverse-transcribed into complementary DNA (cDNA) using the PrimeScript II 1st Strand cDNA Synthesis Kit (Takara Bio, Japan) according to the manufacturer’s instructions. qPCR was performed in a total reaction volume of 20 μL using TB Green Premix Ex Taq II (Takara Bio, Japan) on an ABI StepOnePlus Real-Time PCR System (Applied Biosystems, USA). Relative gene expression was normalized to *β-actin* for mouse liver samples and *GAPDH* for human Huh-7 cells and calculated using the 2^-ΔΔCt^ method. The chow-fed group served as the calibrator for mouse liver analyses, whereas the corresponding untreated or control-transfected group served as the calibrator for cell experiments. Primer sequences are provided in **Supplementary Table 1**. Each biological sample was analyzed in triplicate, and melting-curve analysis was performed to confirm amplification specificity.

### Enzyme-linked immunosorbent assay

Serum FABP5 concentrations measured in chow- and HFHF-fed mice (n = 4 per group) and in patients with MAFLD and healthy controls (n = 20 per group). Blood samples were centrifuged, and the separated serum was collected for analysis. Mouse and human serum FABP5 concentrations were measured using species-specific ELISA kits respectively (Invitrogen, USA) according to the manufacturer’s instructions. Standard curves were generated using the standards supplied with the respective kits, and serum FABP5 concentrations were calculated using CurveExpert Professional software (version 2.6.5; Hyams Development, USA). Each serum sample was assayed in duplicate, and the mean of the two measurements was used for statistical analysis.

### Western blot analysis

Total cellular proteins were extracted on ice for 30 minutes using RIPA lysis buffer supplemented with phenylmethylsulfonyl fluoride (PMSF) and a protease-inhibitor cocktail (Beyotime Biotechnology, China). Protein concentrations were determined using a bicinchoninic acid (BCA) assay kit (Beyotime Biotechnology, China). Protein samples were mixed with SDS loading buffer and denatured at 100 °C for 10 minutes. Equal amounts of protein were separated by sodium dodecyl sulfate–polyacrylamide gel electrophoresis (SDS–PAGE) and transferred onto polyvinylidene fluoride (PVDF) membranes (Millipore, USA).

The membranes were blocked with 5% non-fat milk (BD, USA) for 60 minutes at room temperature and incubated overnight at 4 °C with primary antibodies with primary antibodies against FABP5 (1:500; Abcam, USA) and GAPDH (1:3,000; Abcam, USA). After three washes with Tris-buffered saline containing Tween 20 (TBST; Solarbio, China), the membranes were incubated with the appropriate secondary antibodies for 1 h at room temperature. The membranes were then washed three times with TBST, and immunoreactive bands were visualized using an enhanced chemiluminescence substrate (Solarbio, China) and a ChemiDoc™ XRS+ chemiluminescence imaging system (Bio-Rad Laboratories, USA). FABP5 band intensities were quantified using ImageJ software (National Institutes of Health, USA), and FABP5 expression was normalized to GAPDH expression. Western blot experiments were performed independently three times.

### External validation using public transcriptomic and proteomic datasets

To independently evaluate hepatic *Fabp5*/FABP5 expression in diet-induced fatty liver disease, publicly available transcriptomic and proteomic datasets were reanalyzed. The GSE67678 dataset contains hepatic gene-expression profiles from mice fed a Western diet plus sugar water or a chow diet for 8 weeks. Normalized *Fabp5* expression values were extracted and compared between the two dietary groups.

Hepatic FABP5 protein abundance was further examined using the PXD077557 proteomic dataset, which includes male C57BL/6J mice fed a control diet, high-fat diet, or high-fat/high-fructose diet for 8 weeks. Samples with quantifiable FABP5 abundance were included in the analysis. Protein abundance values were log2-transformed before statistical comparison. Welch’s *t*-test was used to compare the control and HFHFD groups, and proteome-wide multiple-testing correction was performed using the Benjamini–Hochberg method. Both nominal *P* values and false discovery rates were reported.

### Statistical analysis

Statistical analyses were performed using GraphPad Prism version 5.03 (GraphPad Software, USA), unless otherwise specified. Quantitative data are presented as the mean ± standard error of the mean (SEM). Comparisons between two independent groups, including HFHF- versus chow-fed mice and patients with MAFLD versus healthy controls, were performed using two-sided unpaired Student’s *t*-tests. Comparisons among three or more groups were performed using one-way analysis of variance (ANOVA), followed by Tukey’s multiple-comparison test. Cell-based experiments were performed independently three times, and technical replicates within each experiment were averaged before statistical analysis. A two-sided *P* value <0.05 was considered statistically significant.

For the secondary analyses of public datasets, hepatic *Fabp5* expression in GSE67678 and hepatic FABP5 protein abundance in PXD077557 were compared using two-sided Welch’s *t*-tests. For the PXD077557 proteomic analysis, nominal *P* values were adjusted across 8,261 protein entries using the Benjamini–Hochberg procedure. An FDR <0.05 was considered statistically significant after multiple-testing correction. The proteomic abundance values were transformed as log_2_(abundance + 1) before statistical analysis.

## Results

### HFHF feeding induces hepatic injury and global transcriptomic alterations in mice

**Figure 1:**
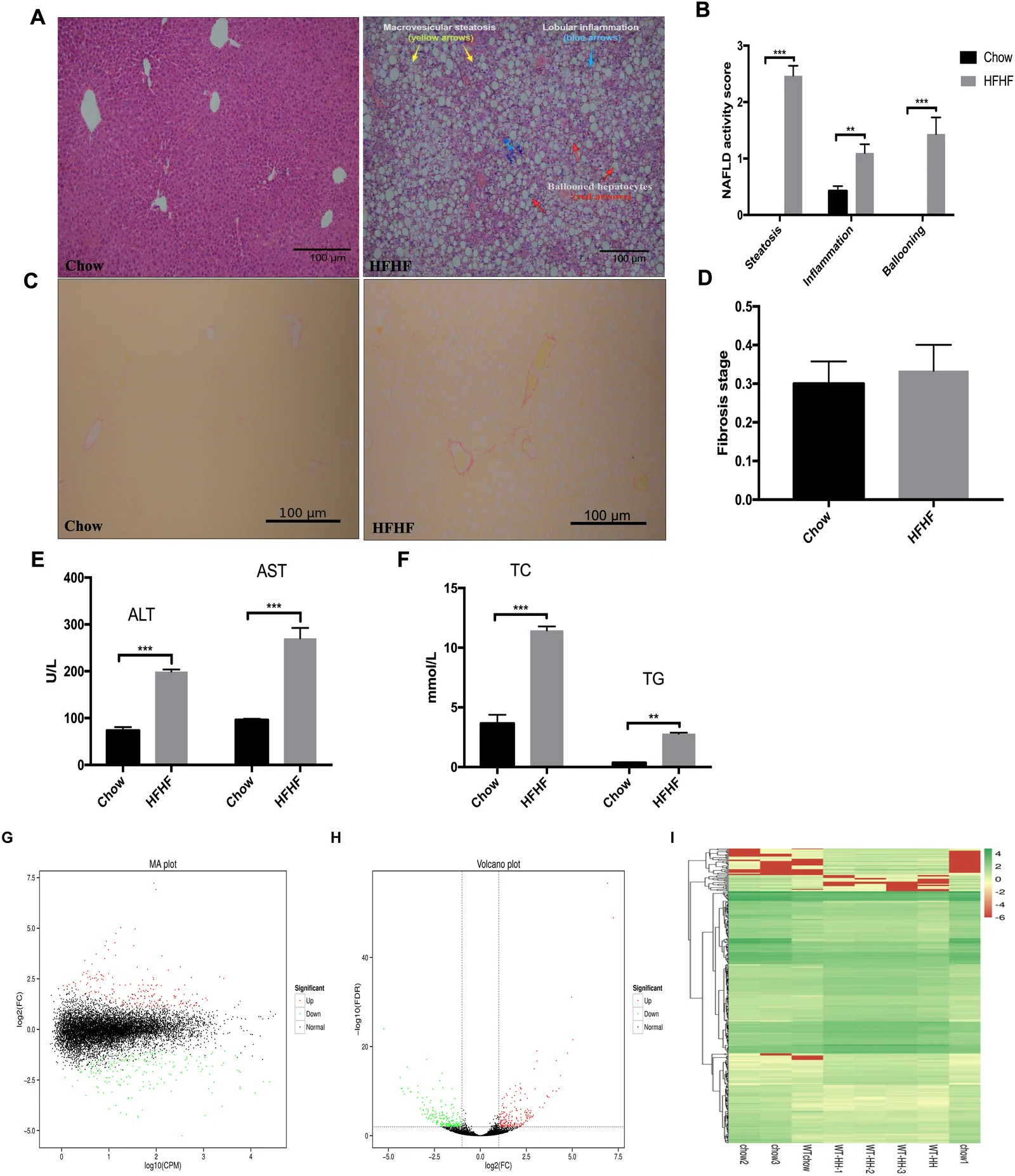
HFHF feeding induces hepatic injury, metabolic abnormalities, and global transcriptomic alterations in mice. **(A)** Representative hematoxylin and eosin-stained liver sections from chow- and HFHF-fed mice. Yellow, blue, and red arrows indicate macrovesicular steatosis, lobular inflammatory foci, and ballooned hepatocytes, respectively. Scale bars, 100 μm. **(B)** Histological scores for hepatic steatosis, lobular inflammation, and hepatocellular ballooning. **(C)** Representative sirius red-stained liver sections. Scale bars, 100 μm. **(D)** Histological assessment of hepatic fibrosis. **(E)** Serum alanine aminotransferase (ALT) and aspartate aminotransferase (AST) concentrations. **(F)** Serum total cholesterol (TC) and triglyceride (TG) concentrations. **(G)** MA plot showing global gene-expression changes between HFHF- and chow-fed mice. **(H)** Volcano plot of differentially expressed genes. Red and green dots indicate significantly upregulated and downregulated genes, respectively. **(I)** Hierarchical-clustering heatmap of the differentially expressed genes. Data in panels B and D–F are presented as the mean ± SEM (*n* = 4 mice per group). Statistical comparisons were performed using two-sided unpaired Student’s *t*-tests. **P < 0.01; ***P < 0.001.

After 19 weeks of feeding, HFHF-fed mice exhibited marked hepatic steatosis compared with chow-ded controls. H&E staining demonstrated macrovesicular steatosis (yellow arrows), lobular inflammatory cell infiltration (blue arrows), and hepatocellular ballooning (red arrows) in the HFHF group, whereas normal hepatic architecture was largely preserved in the chow group (**Figure 1A**). To quantitatively assess these histopathological changes, liver injury was scored using the NAFLD activity score (NAS) system (30). Consistently, the steatosis, lobular inflammation, and hepatocellular ballooning were significantly higher in HFHF-fed mice (P < 0.01 or P < 0.001 as indicated, n=4, **Figure 1B**). No significant hepatic fibrosis was detected in either group at this stage (**Figures 1C, D**).

Serum alanine aminotransferase (ALT) and aspartate aminotransferase (AST) concentrations were significantly elevated in HFHF-fed mice, indicating the presence of hepatocellular injury (P <0.001, n=4, **Figure 1E**). Serum total cholesterol (TC) and triglyceride (TG) concentrations were also significantly higher in the HFHF group than in the chow group (P < 0.01 or P < 0.001 as indicated, n=4, **Figure 1F**), consistent with systemic lipid metabolic dysfunction.

To characterize hepatic transcriptional alterations associated with HFHF feeding, ONT transcriptomic sequencing was performed on liver tissues collected from HFHF- and chow-fed mice. A total of 400 differentially expressed genes (DEGs) were identified including 201 up-regulated and 199 down-regulated in the HFHF-fed mice (**Table 1**; **Supplementary Table 2**). Among these, 13 were long non-coding RNAs (LncRNAs), comprising 8 upregulated and 5 downregulated genes, whereas 387 were protein-coding mRNAs, comprising 193 upregulated and 194 downregulated genes. The MA and volcano plots showed widespread transcriptional changes following HFHF feeding (**Figures 1G, H**). Hierarchical clustering based on the DEGs separated the HFHF and chow samples into distinct groups, indicating consistent differences in their hepatic transcriptomic profiles (**Figure 1I**).

**Table 1:** The differentially expressed genes between HFHF group and Chow group.

Gene description	DEG number	Up-regulated	Down-regulated
Total RNAs	400	201	199
LncRANs	13	8	5
mRNAs	387	193	194

### Functional enrichment identifies 12 differentially expressed genes involved in lipid transport and metabolism

**Figure 2:**
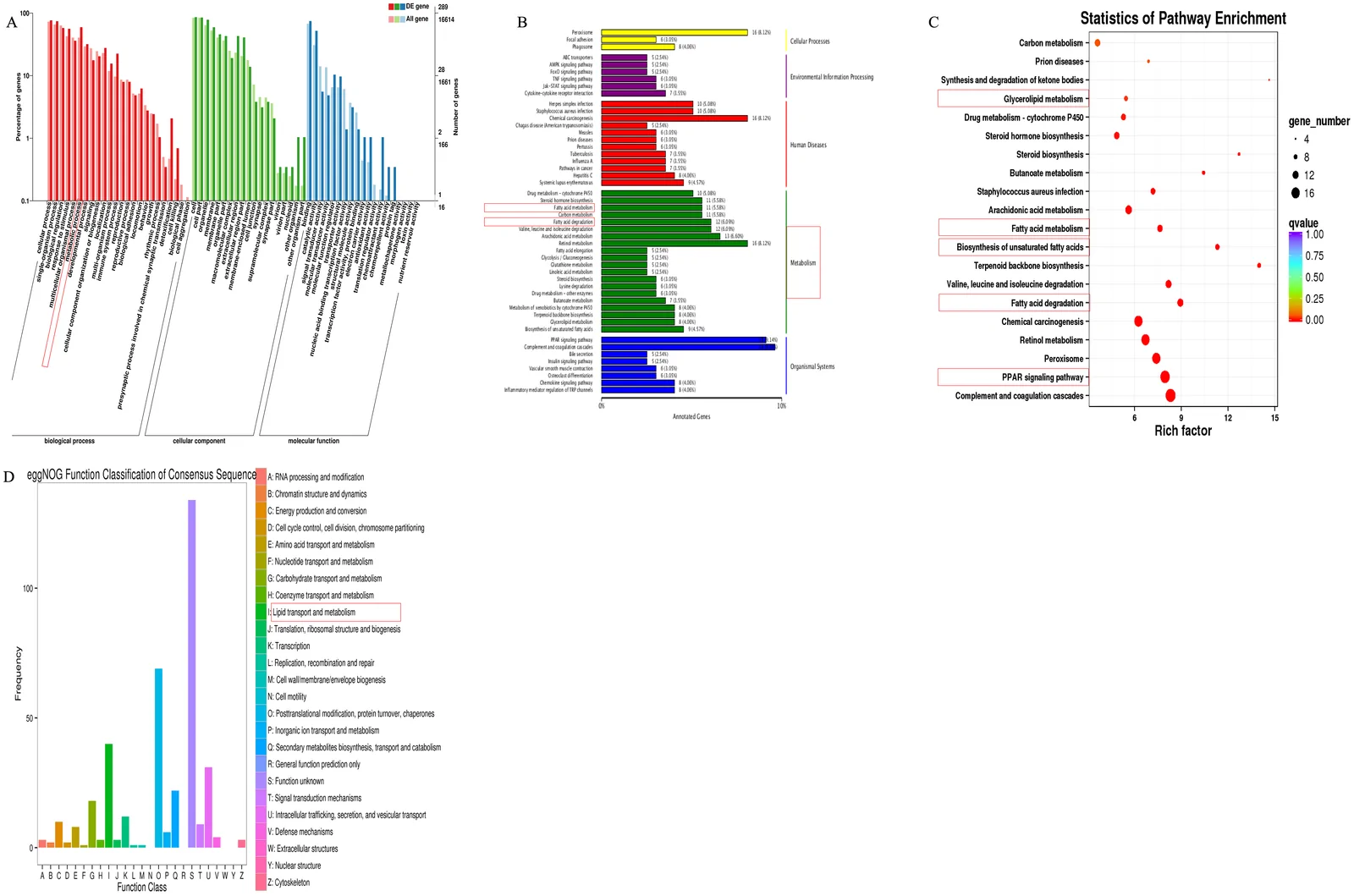
Functional annotation and enrichment analysis of differentially expressed genes in the livers of HFHF- and chow-fed mice. **(A)** Gene ontology (GO) classification of the differentially expressed genes (DEGs) according to biological process, cellular component, and molecular function. Several GO categories were associated with metabolic regulation. **(B)** Broad Kyoto Encyclopedia of Genes and Genomes (KEGG) pathway classification of the DEGs. **(C)** KEGG enrichment analysis showing the top 20 significantly enriched pathways. Lipid metabolism-related pathways are highlighted by red circles. Pathways with a false discovery rate-adjusted *q* value <0.05 were considered significantly enriched. **(D)** eggNOG functional classification of the protein-coding DEGs. Genes assigned to functional category I, “lipid transport and metabolism,” were subsequently extracted to identify the 12 candidate genes presented in **Table 2** and validated by RT-qPCR in **Figure 3**.

Gene Ontology (GO) analysis showed that the DEGs were distributed across multiple biological processes, cellular components, and molecular functions, including several categories associated with metabolic regulation (**Figure 2A**). KEGG analysis demonstrated enrichment of pathways related to fatty acid metabolism, fatty acid degradation, biosynthesis of unsaturated fatty acids, glycerolipid metabolism, peroxisomal function, and PPAR signaling (**Figures 2B, C**). In addition, eggNOG functional classification assigned a subset of the protein-coding DEGs to the “lipid transport and metabolism” category (**Figure 2D**).

Among the protein-coding DEGs meeting the prespecified differential-expression criteria, 12 genes were assigned to the eggNOG “lipid transport and metabolism” functional category. These comprised eight upregulated and four downregulated genes in HFHF-fed mice (**Table 2**; **Supplementary Table 2**). RT-qPCR analysis confirmed significant upregulation of *Mogat1*, *Osbpl3*, *Acss3*, *Acaa1b*, *Acot11*, *Fitm1*, *Fabp2*, and *Ehhadh*, and significant downregulation of *Idi1*, *Isyna1*, *Fabp5*, and *Sqle* in HFHF-fed mice (**Figure 3**). The direction of change for all 12 genes was consistent with the ONT transcriptomic result.

**Table 2:** The differentially expressed genes involved in lipid transport and metabolism between HFHF group and Chow group.

#ID	Gene_type	Symbol	FDR	log2FC	Regulated
ENSMUSG00000012187	Known_gene	Mogat1	3.03E-22	5.029984826	up
ENSMUSG00000029822	Known_gene	Osbpl3	7.23E-05	2.763234689	up
ENSMUSG00000035948	Known_gene	Acss3	6.02E-06	2.718040597	up
ENSMUSG00000010651	Known_gene	Acaa1b	2.07E-05	2.596536491	up
ENSMUSG00000034853	Known_gene	Acot11	6.19E-06	2.494084202	up
ENSMUSG00000022215	Known_gene	Fitm1	1.97E-05	2.472352965	up
ENSMUSG00000023057	Known_gene	Fabp2	1.22E-06	2.19519916	up
ENSMUSG00000022853	Known_gene	Ehhadh	2.82E-10	2.181263814	up
ENSMUSG00000058258	Known_gene	Idi1	9.31E-06	-2.231489447	down
ENSMUSG00000019139	Known_gene	Isyna1	1.79E-10	-3.382855844	down
ENSMUSG00000027533	Known_gene	Fabp5	7.10E-12	-3.404935009	down
ENSMUSG00000022351	Known_gene	Sqle	3.93E-16	-3.985599723	down

**Figure 3:**
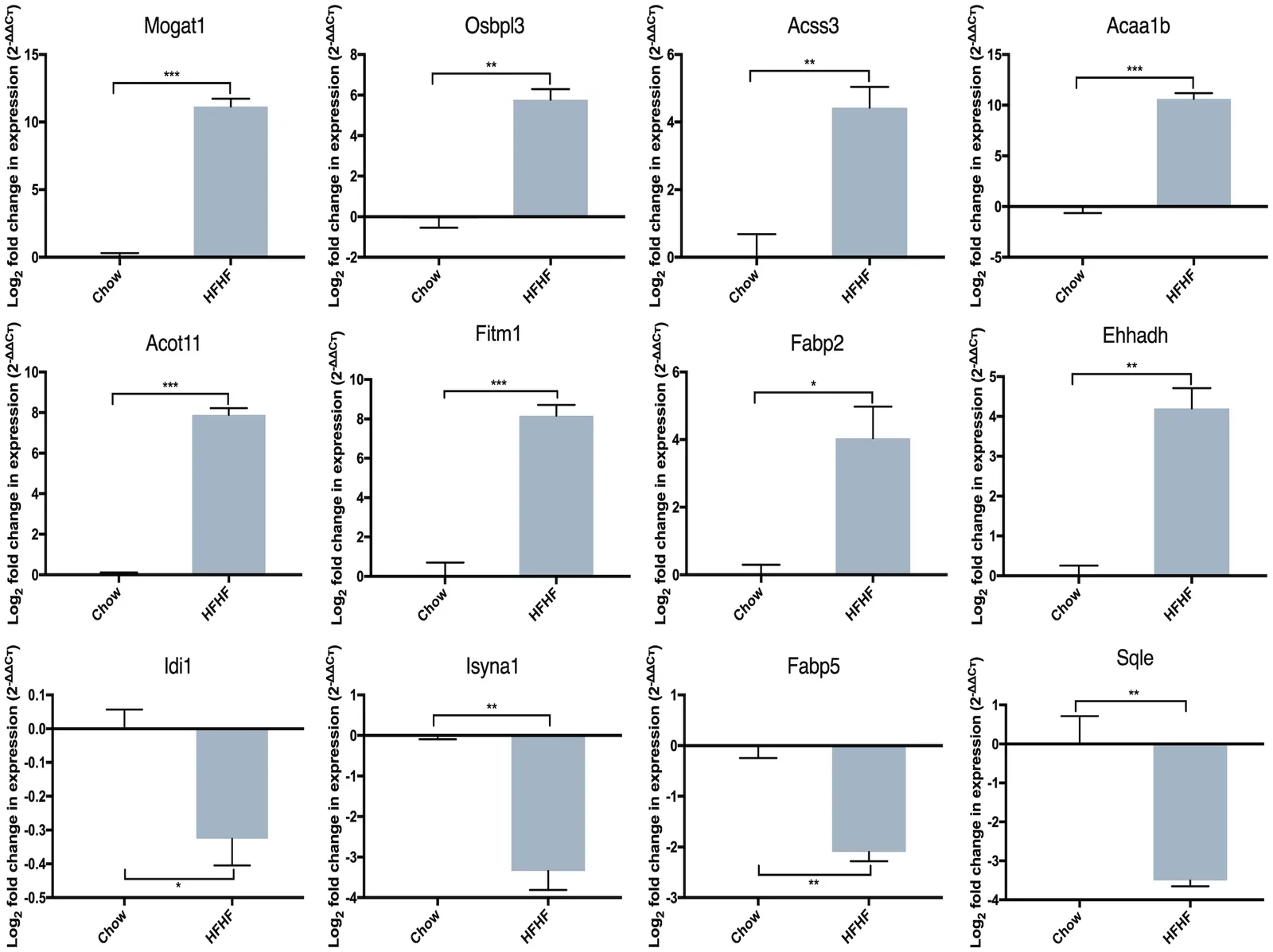
RT-qPCR validation of the 12 differentially expressed genes involved in lipid transport and metabolism. Hepatic expression of the 12 candidate genes identified by ONT transcriptomic sequencing and eggNOG functional classification was measured by RT-qPCR in chow- and HFHF-fed mice. *Mogat1*, *Osbpl3*, *Acss3*, *Acaa1b*, *Acot11*, *Fitm1*, *Fabp2*, and *Ehhadh* were significantly upregulated, whereas *Idi1*, *Isyna1*, *Fabp5*, and *Sqle* were significantly downregulated in HFHF-fed mice. The direction of change for all 12 genes was consistent with the ONT transcriptomic results. Gene expression was normalized to *β-actin*, and the chow group was used as the calibrator. Data are presented as the mean ± SEM (*n* = 4 biologically independent mice per group). Statistical comparisons were performed using two-sided unpaired Student’s *t*-tests. *P < 0.05, **P < 0.01, and ***P < 0.001.

### Circulating FABP5 concentrations are reduced in HFHF-fed mice and patients with MAFLD

**Figure 4:**
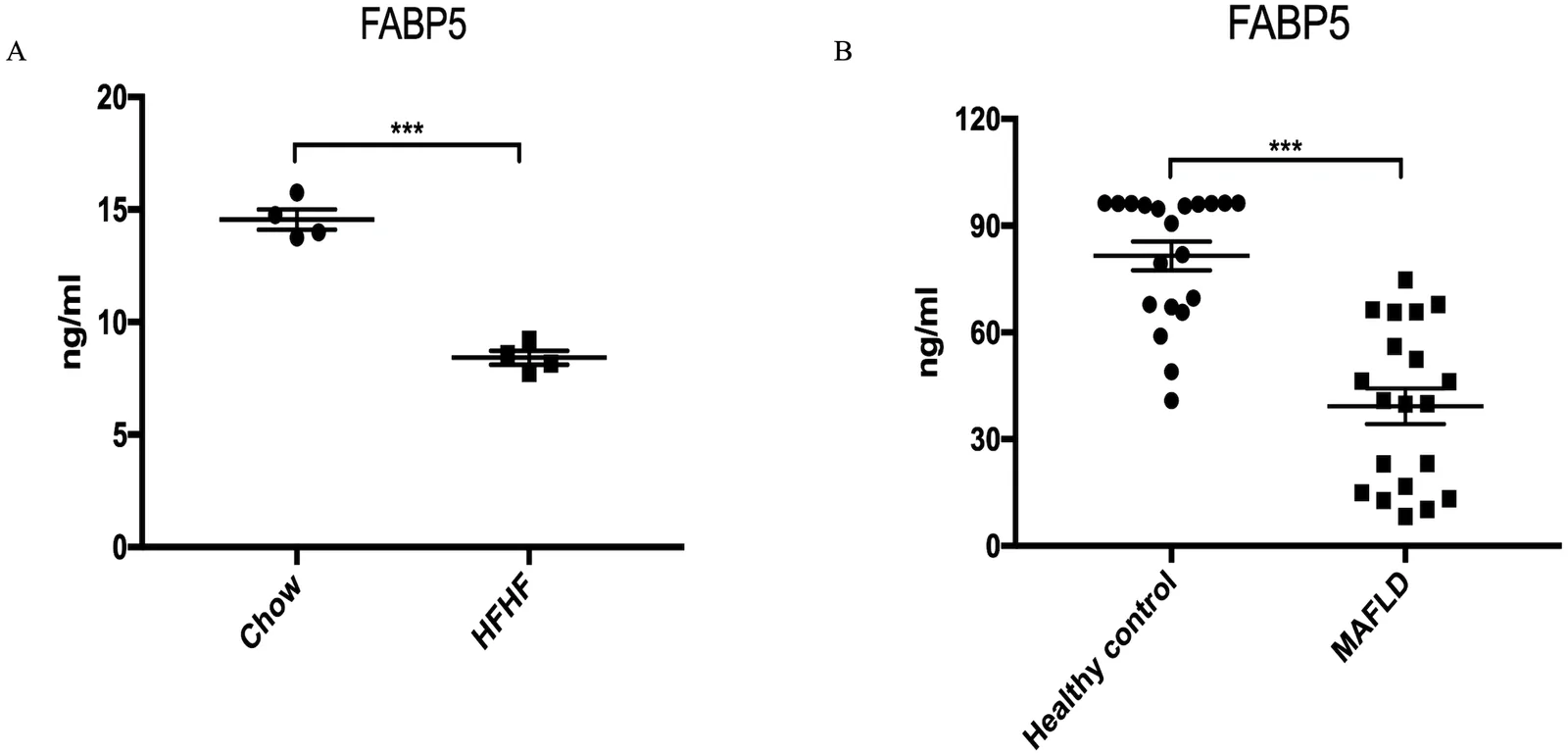
Serum FABP5 concentrations in HFHF-fed mice and patients with MAFLD. **(A)** Serum FABP5 concentrations were measured by ELISA in chow- and HFHF-fed mice. FABP5 concentrations were significantly lower in HFHF-fed mice than in chow-fed controls (*n* = 4 biologically independent mice per group). **(B)** Serum FABP5 concentrations were measured in patients with MAFLD and healthy controls. FABP5 concentrations were significantly lower in patients with MAFLD than in healthy controls (*n* = 20 participants per group). Data are presented as the mean ± SEM. Statistical comparisons were performed using two-sided unpaired student’s *t*-tests. ***P < 0.001.

Among the 12 lipid transport- and metabolism-related DEGs, *Fabp5* was selected for further investigation because its hepatic expression was significantly reduced in HFHF-fed mice, FABP5 is involved in intracellular fatty acid trafficking, and the protein can be measured in the circulation. Serum FABP5 concentrations were significantly lower in HFHF-fed mice than in chow-fed controls (**Figure 4A**, n=4 per group).

To examine whether this finding was also observed in human MAFLD, serum FABP5 concentrations were measured in patients with MAFLD and healthy controls. The clinical characteristics of the participants were summarized in **Table 3**. Serum FABP5 concentrations were significantly lower in patients with MAFLD than in healthy controls (**Figure 4B**, n=20 per group), consistent with the direction of change observed in HFHF-fed mice.

**Table 3:** Details of patients with MAFLD.

			Hepatic steatosis				Metabolic abnormalities		
No.	Age (years)	Gender	Ultrasound	MRI	Overweight (BMI≥23 Kg/m^2^)	Type 2 diabetes mellitus	High blood pressure (≥130/85 mmHg)	High triglycerides (≥1.7mmol/L)	Low HDL-c (male<1.0mmol/L,female<1.3mmol/L)
**1**	42	female	✓		✓				
**2**	47	female	✓					✓	✓
**3**	63	female	✓		✓				
**4**	72	female	✓		✓				
**5**	44	male	✓		✓				
**6**	60	female	✓		✓				
**7**	64	male	✓		✓				
**8**	50	female	✓		✓				
**9**	59	male	✓		✓				
**10**	33	male	✓			✓			
**11**	55	male	✓				✓	✓	
**12**	43	female	✓				✓		✓
**13**	63	female	✓		✓				
**14**	43	female	✓		✓				
**15**	63	male	✓		✓				
**16**	30	male		✓	✓				
**17**	56	male	✓			✓			
**18**	41	female	✓		✓				
**19**	36	male	✓				✓		✓
**20**	74	female	✓		✓				

### Hepatic FABP5 immunoreactivity decreases during MCD diet-induced steatosis

**Figure 5:**
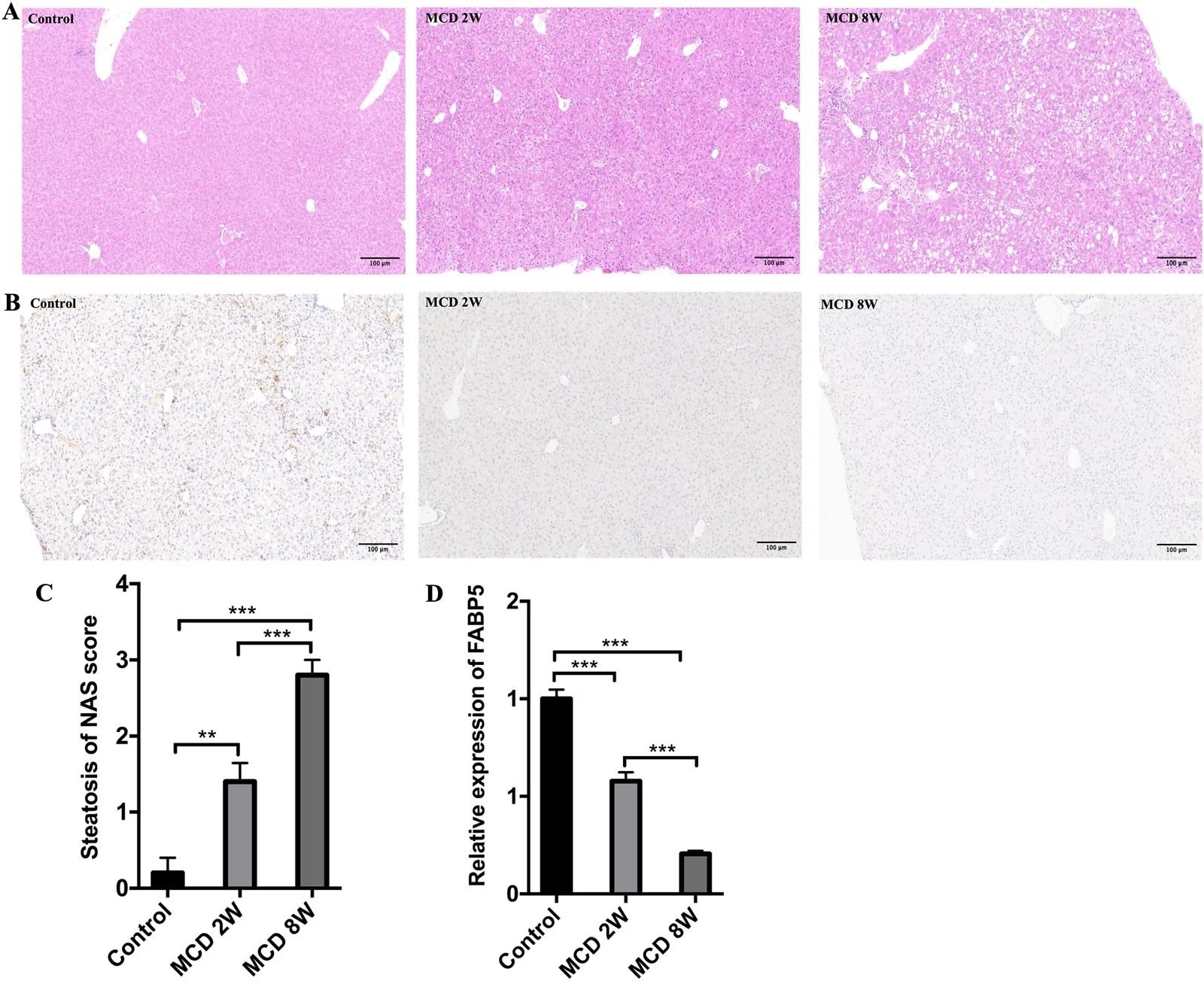
Hepatic FABP5 immunoreactivity decreases during MCD diet-induced steatosis. Wild-type C57BL/6N mice were fed a methionine- and choline-deficient (MCD) diet for 2 or 8 weeks, while mice fed a chow diet for 8 weeks served as controls. **(A)** Representative hematoxylin and eosin (H&E)-stained liver sections showing hepatic steatosis in the control, 2-week MCD, and 8-week MCD groups. Original magnification, ×100. **(B)** Representative immunohistochemical staining of FABP5 in liver sections from the three groups. Original magnification, ×100. **(C)** Histological steatosis scores assessed according to the NAFLD activity score system. **(D)** Quantification of hepatic FABP5 immunoreactivity using ImageJ software. Data are presented as the mean ± SEM (*n* = 5 biologically independent mice per group). Statistical comparisons were performed using one-way ANOVA followed by Tukey’s multiple-comparison test. **P < 0.01 and ***P < 0.001.

MCD feeding induced substantial hepatic steatosis at both 2 and 8 weeks, with a greater steatosis score at 8 weeks than at 2 weeks (**Figures 5A, C**). Immunohistochemical analysis demonstrated reduced hepatic FABP5 immunoreactivity after 2 weeks of MCD feeding and a further reduction after 8 weeks (**Figures 5B, D**). Quantification of the staining confirmed significantly lower FABP5 immunoreactivity in both MCD groups than in controls.

### Independent public datasets support reduced hepatic Fabp5/FABP5 abundance in diet-induced fatty liver models

**Figure 6:**
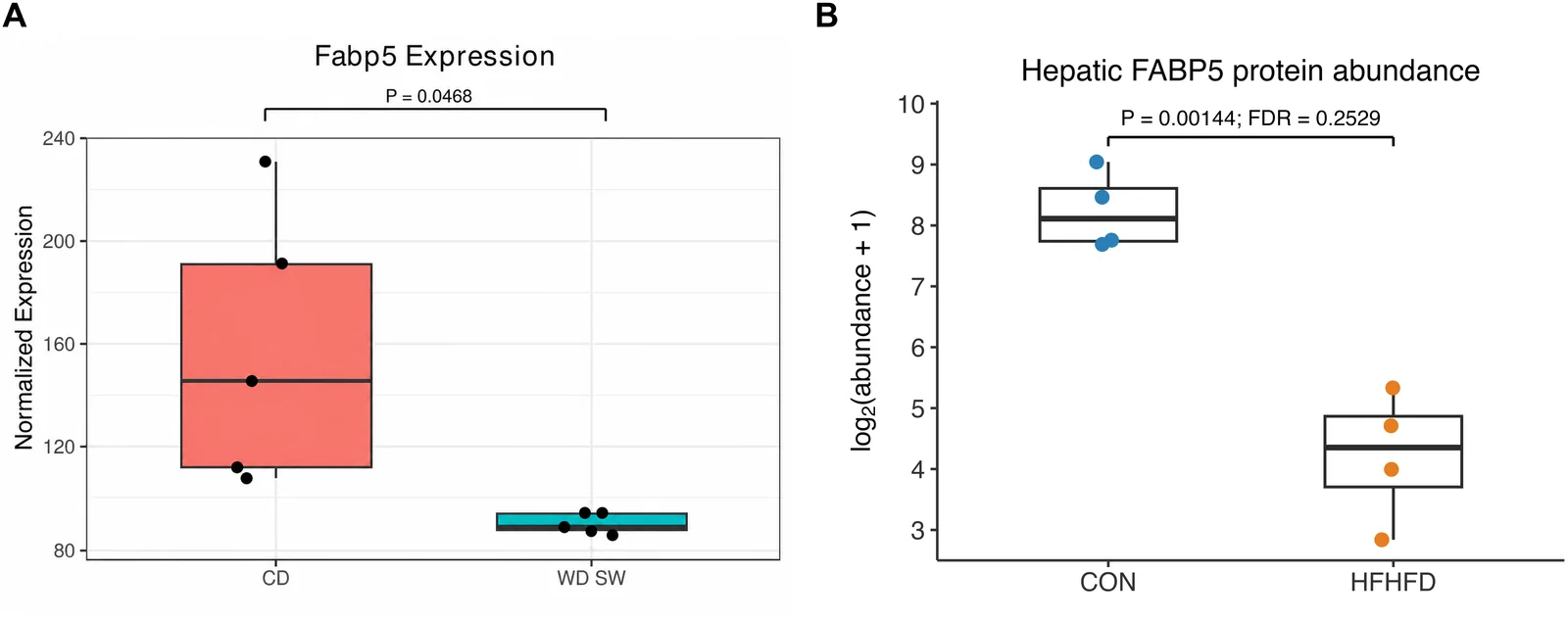
Independent transcriptomic and proteomic analyses of hepatic *Fabp5*/FABP5 expression in diet-induced fatty liver models. **(A)** Hepatic *Fabp5* mRNA expression in the GSE67678 dataset. Normalized *Fabp5* expression was compared between mice fed a chow diet (CD) and mice fed a Western diet plus fructose-glucose-containing sugar water (WD/SW) for 8 weeks. For each sample, the normalized expression values of two *Fabp5* probes (ILMN_1235908 and ILMN_2685290) were averaged. Each point represents one mouse (n = 5 per group). Boxes indicate the median and interquartile range, and whiskers represent the data range. Statistical significance was assessed using a two-sided Welch’s *t*-test (*P* = 0.0468). **(B)** Hepatic FABP5 protein abundance in the PXD077557 dataset. FABP5 abundance was compared between mice fed a control diet (CON) and those fed a high-fat/high-fructose diet (HFHFD). Protein abundance values were transformed as log_2_(abundance + 1) before statistical analysis. Each point represents one mouse (n = 4 per group). Boxes indicate the median and interquartile range, and whiskers extend to the most extreme values within 1.5 times the interquartile range. Nominal significance was assessed using a two-sided Welch’s *t*-test, followed by Benjamini–Hochberg correction across 8,261 protein entries. Hepatic FABP5 abundance was lower in the HFHFD group than in the CON group (nominal *P* = 0.00144; FDR = 0.2529), although the difference did not remain significant after proteome-wide multiple-testing correction.

To assess whether the reduction in hepatic *Fabp5* observed in our HFHF-fed mice could be reproduced in independent datasets, we analyzed publicly available hepatic transcriptomic and proteomic data. In the GSE67678 dataset, hepatic *Fabp5* mRNA expression was lower in mice fed a Western diet supplemented with fructose–glucose-containing sugar water (WD/SW) for 8 weeks than in chow-fed controls (two-sided Welch’s *t*-test, *P* = 0.0468; **Figure 6A**). This finding was directionally consistent with both the ONT sequencing and RT-qPCR results obtained in our HFHF-fed mice.

We next examined hepatic protein abundance in the PXD077557 proteomic dataset. FABP5 abundance was markedly lower in HFHFD-fed mice than in control-diet-fed mice (mean abundance: 21.39 versus 324.81; log_2_ fold change = −4.019; nominal *P* = 0.00144; **Figure 6B**). However, this difference did not remain statistically significant after Benjamini–Hochberg correction across 8,261 protein entries (FDR = 0.2529). Collectively, these independent datasets provide supportive cross-platform evidence for reduced hepatic FABP5 abundance in diet-induced fatty liver models, but they do not constitute direct protein-level validation in our original 19-week HFHF cohort.

### PA induces lipid accumulation and alters the expression of fatty acid oxidation-related genes in Huh-7 cells

**Figure 7:**
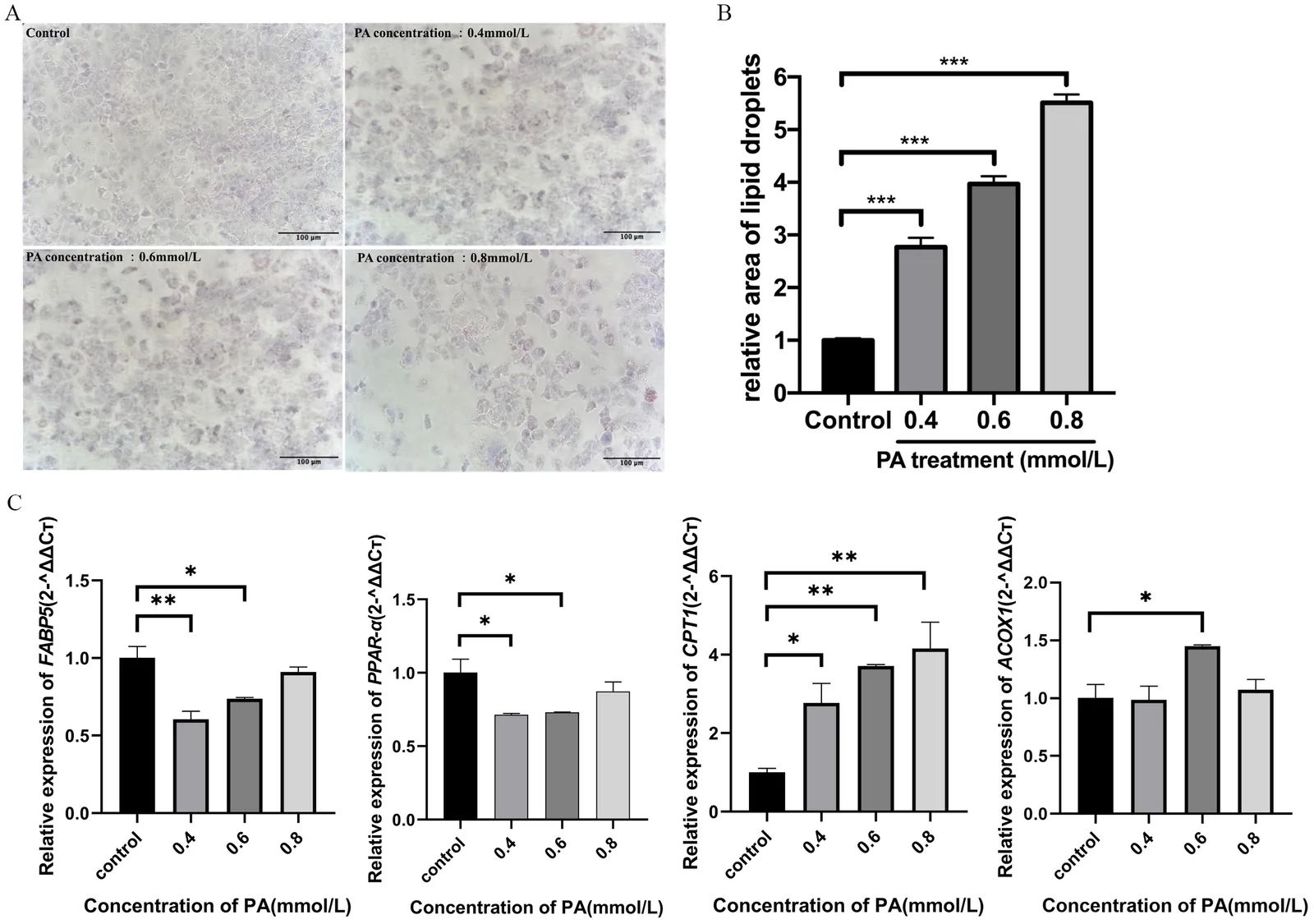
PA induces intracellular lipid accumulation and alters the expression of fatty acid oxidation-related genes in Huh-7 cells. Huh-7 cells were treated with vehicle control, 0.4, 0.6, or 0.8 mmol/L palmitic acid (PA) for 24 h. **(A)** Representative images of Oil Red O staining showing intracellular lipid accumulation following treatment with the indicated PA concentrations. **(B)** Quantification of the relative Oil Red O-positive lipid-droplet area using ImageJ software. **(C)** Relative mRNA expression of *FABP5*, *PPAR-α*, *CPT1A*, and *ACOX1*, as determined by RT-qPCR. Gene expression was normalized to *GAPDH*, and the untreated control group was used as the calibrator. Data are presented as the mean ± SEM from three independent experiments (*n* = 3). Statistical comparisons were performed using one-way ANOVA followed by followed by Dunnett’s multiple-comparison test. *P < 0.05, **P < 0.01, and ***P < 0.001.

Oil Red O staining demonstrated that treatment with 0.4, 0.6, or 0.8 mmol/L PA for 24 h increased intracellular lipid accumulation in Huh-7 cells compared with untreated controls. Lipid droplet accumulation increased in a concentration-dependent manner (**Figures 7A, B**).

PA treatment also altered the expression of genes involved in fatty acid metabolism. Compared with vehicle control, *FABP5* and *peroxisome proliferator-activated receptor-α* (*PPAR-α*) mRNA expression was significantly reduced followed treatment with 0.4 and 0.6 mmol/L PA. By contrast, the mRNA expression of *Carnitine Palmitoyl transferase 1* (*CPT1*) was increased at the tested PA concentrations, whereas *Acyl Coenzyme A Oxidase 1* (*ACOX1*) expression was significantly increased at 0.6 mmol/L PA (**Figure 7C**). Based on the induction of lipid accumulation and the accompanying reduction in *FABP5* expression, 0.6 mmol/L PA was selected for the subsequent FABP5-overexpression experiments.

### FABP5 overexpression attenuates PA-induced lipid accumulation and is associated with increased expression of fatty acid oxidation-related genes

**Figure 8:**
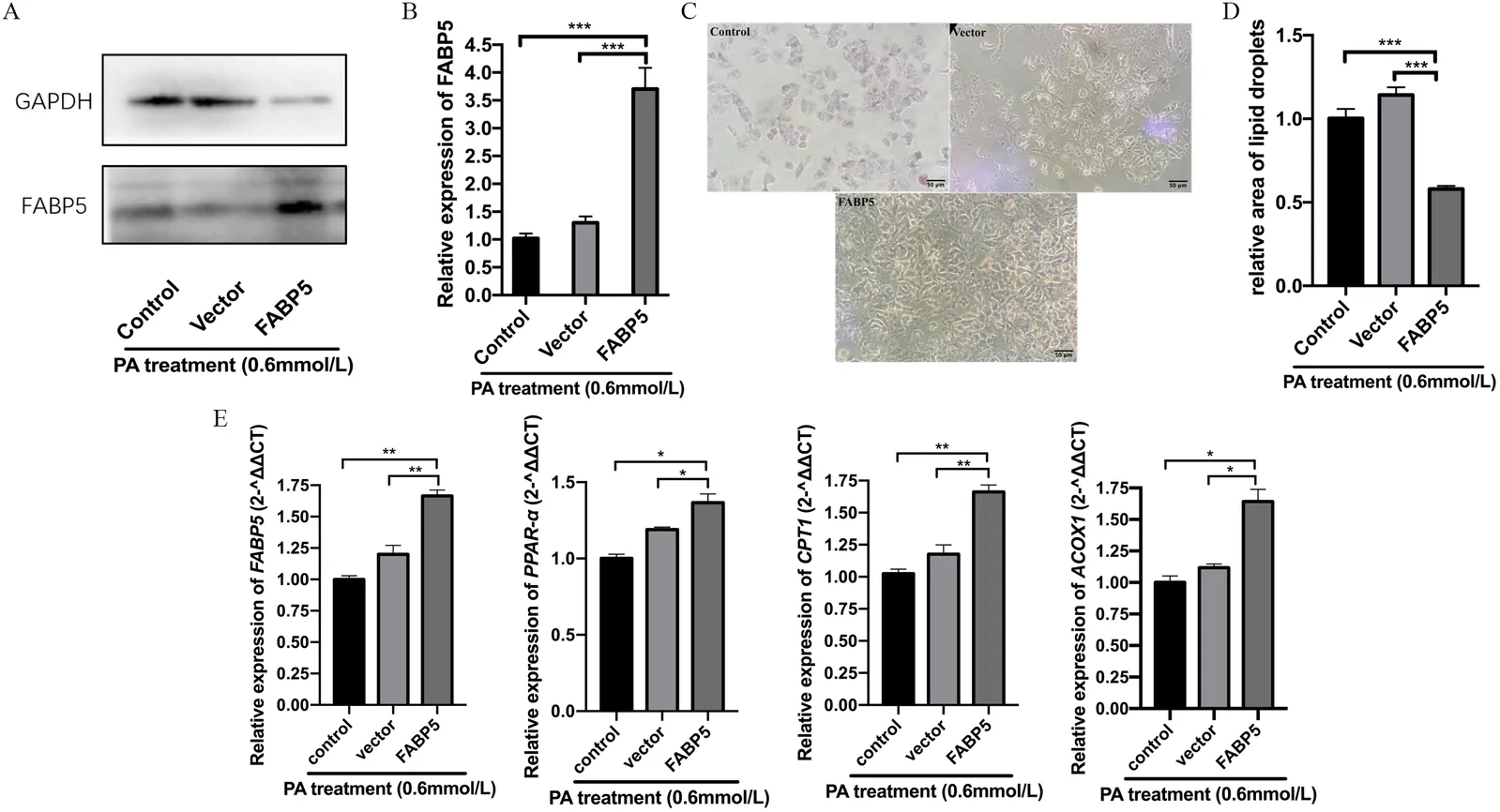
FABP5 overexpression attenuates PA-induced lipid accumulation and is associated with increased expression of fatty acid oxidation-related genes in Huh-7 cells. Huh-7 cells were left untransfected (Control), transfected with the empty vector (Vector), or transfected with the FABP5 expression plasmid (FABP5 OE), followed by treatment with 0.6 mmol/L palmitic acid (PA) for 24 h. **(A)** Western blot analysis of FABP5 protein abundance in the indicated groups. GAPDH was used as the loading control. **(B)** Densitometric quantification of FABP5 protein abundance using ImageJ, with FABP5 band intensity normalized to GAPDH. **(C)** Representative Oil Red O-stained images showing intracellular lipid accumulation in the indicated groups. **(D)** Quantification of the relative Oil Red O-positive lipid-droplet area using ImageJ. **(E)** Relative mRNA expression of *FABP5*, *PPAR-α*, *CPT1A*, and *ACOX1*, as determined by RT-qPCR. Gene expression was normalized to *GAPDH*, and the control group was used as the calibrator. Data are presented as the mean ± SEM from three independent experiments (*n* = 3). Statistical comparisons were performed using one-way ANOVA followed by Tukey’s multiple-comparison test. *P < 0.05, **P < 0.01, and ***P < 0.001.

FABP5 overexpression in Huh-7 cells was confirmed by immunoblotting and RT-qPCR. FABP5 protein and mRNA levels were significantly higher in FABP5-plasmid-transfected cells than in control and empty-vector-transfected cells (**Figures 8A, B, E**).

Following treatment with 0.6 mmol/L PA for 24 h, Oil Red O staining demonstrated significantly lower intracellular lipid accumulation in FABP5-overexpressing cells than in control and empty-vector-transfected cells (**Figures 8C, D**). FABP5 over-expression was also accompanied by significantly increased mRNA expression of *PPAR-α*, *CPT1* and *ACOX1* (**Figure 8E**). These findings indicate that FABP5 overexpression attenuates PA-induced lipid accumulation and is associated with changes in the expression of genes involved in fatty acid oxidation.

## Discussion

In the current study, ONT-based hepatic transcriptomic profiling identified 400 differentially expressed genes in mice fed an HFHF diet for 19 weeks. Functional classification further identified 12 protein-coding genes involved in lipid transport and metabolism, and the direction of change for all 12 genes was confirmed by RT-qPCR. Among these genes, hepatic *Fabp5* expression was reduced in HFHF-fed mice. This reduction was accompanied by lower circulating FABP5 concentrations in both HFHF-fed mice and patients with MAFLD. Reduced hepatic FABP5 immunoreactivity was also observed in an independent MCD diet model. Furthermore, analyses of publicly available transcriptomic and proteomic datasets showed directionally consistent reductions in hepatic *Fabp5* mRNA and FABP5 protein abundance in other diet-induced fatty liver models. Functionally, FABP5 overexpression attenuated PA-induced lipid accumulation in Huh-7 cells and was associated with increased expression of *PPAR-α*, *CPT1*, and *ACOX1*. Collectively, these findings identify FABP5 as a candidate regulator associated with hepatic lipid metabolism, although they do not yet establish FABP5 as a validated biomarker or therapeutic target for MAFLD.

The 12 candidate genes were selected using a predefined bioinformatic procedure rather than by subjective selection. Specifically, protein-coding genes meeting the differential-expression criteria in the HFHF-versus-chow comparison were intersected with the eggNOG “lipid transport and metabolism” functional category. This procedure identified eight upregulated genes—*Mogat1*, *Osbpl3*, *Acss3*, *Acaa1b*, *Acot11*, *Fitm1*, *Fabp2*, and *Ehhadh*—and four downregulated genes—*Idi1*, *Isyna1*, *Fabp5*, and *Sqle*. Previous studies have implicated several of these genes in triglyceride synthesis, lipid-droplet formation, sterol metabolism, and mitochondrial or peroxisomal fatty acid oxidation (31–37). The agreement between the ONT and RT-qPCR results supports the technical reproducibility of the transcriptomic findings. Nevertheless, these genes were identified in a relatively small exploratory cohort, and their individual contributions to MAFLD pathogenesis require validation in additional experimental models.

FABP5 was prioritized for further investigation because it functions as an intracellular lipid chaperone that binds long-chain fatty acids and regulates their intracellular trafficking and delivery to metabolic and nuclear targets (16–21, 25, 38). FABP5 can also be detected in the circulation and circulating FABP5 concentrations have previously been associated with type 2 diabetes, dyslipidemia, atherosclerosis, and other metabolic abnormalities (27–29). However, FABP5 is primarily a cytosolic protein and should not be classified as a conventionally secreted protein solely because it is detectable in serum. Its presence in the circulation may result from non-classical secretion, extracellular-vesicle release, cellular turnover, or tissue injury, and may originate from several tissues rather than exclusively from the liver (17–19, 38–40). Consequently, the lower serum FABP5 concentrations observed in HFHF-fed mice and patients with MAFLD cannot be assumed to directly reflect hepatic or hepatocyte FABP5 abundance. Moreover, the human analysis included only 20 patients with MAFLD and 20 healthy controls and did not assess diagnostic performance or adjust for potential metabolic confounders. Our findings therefore demonstrate an association between circulating FABP5 and MAFLD but are insufficient to establish FABP5 as a diagnostic biomarker.

Our observation of reduced hepatic *Fabp5* expression differs from that of Westerbacka et al., who reported approximately 2.5-fold higher hepatic FABP5 mRNA expression in patients with nonalcoholic fatty liver and found a positive association between FABP5 expression and liver fat content (41). Several factors may explain this apparent discrepancy. First, the studies differed in species, dietary composition, duration of exposure, disease stage, and metabolic background. The previous study analyzed bulk-liver samples from insulin-resistant human participants with variable degrees of hepatic steatosis, whereas our primary transcriptomic analysis was performed in mice after 19 weeks of HFHF feeding (41). Second, FABP5 expression may change dynamically during the progression from simple steatosis to steatohepatitis and fibrosis. The GSE67678 model, for example, develops steatosis at approximately 4–8 weeks, steatohepatitis at 12–16 weeks, progressive fibrosis after 16 weeks, and hepatocellular carcinoma at later stages (42). In our secondary analysis of the 8-week GSE67678 samples, hepatic *Fabp5* mRNA expression was lower in WD/SW-fed mice, supporting the possibility that reduced expression can occur during the predominantly steatotic stage. Third, differences in the cellular composition of liver specimens may contribute to apparently conflicting results. Bulk-liver transcriptomic analysis represents the average expression across hepatocytes and multiple non-parenchymal cell populations. FABP5 is abundantly expressed in macrophages and can regulate their inflammatory and metabolic phenotypes (17, 38, 43). Recent single-cell studies have also identified FABP5-high macrophage populations in advanced liver fibrosis and carcinogenesis (44). Therefore, increased FABP5 expression in a relatively small population of lipid-associated or fibrosis-associated macrophages could coexist with reduced expression in hepatocytes or other abundant hepatic cell populations. Changes in disease stage and cellular composition could consequently produce opposing results between bulk-tissue, isolated-cell, and single-cell analyses.

The reduction in hepatic *Fabp5* may represent an adaptive response to chronic lipid overload that limits fatty acid trafficking or FABP5-dependent inflammatory signaling. Alternatively, reduced FABP5 could impair intracellular fatty acid buffering and metabolic trafficking, thereby favoring lipid storage rather than oxidative utilization. The latter interpretation is compatible with our observation that FABP5 overexpression reduced PA-induced lipid accumulation in Huh-7 cells. However, these possibilities are not mutually exclusive. FABP5 function depends on the expressing cell type, ligand availability, intracellular localization, and metabolic or inflammatory state (17, 18, 25, 38). For example, FABP5 deficiency can alter macrophage inflammatory responses through AMPK/NF-κB-related mechanisms, illustrating that its biological effects extend beyond hepatocyte lipid oxidation (45). Thus, FABP5 may exert distinct or even opposing effects in hepatocytes and hepatic macrophages.

The available protein-level evidence should also be interpreted cautiously. Hepatic FABP5 protein expression could not be examined by immunoblotting or immunohistochemistry in the original 19-week HFHF cohort because suitable liver specimens were no longer available. Nevertheless, reduced hepatic FABP5 immunoreactivity was observed during MCD diet-induced steatosis, and secondary analysis of the independent PXD077557 dataset demonstrated markedly lower hepatic FABP5 protein abundance in HFHFD-fed mice. Although the difference in PXD077557 was nominally significant, it did not remain significant after Benjamini–Hochberg correction across 8,261 protein entries. The public proteomic result therefore provides supportive exploratory evidence rather than statistically definitive protein-level validation.

In addition, the MCD and HFHF models represent different metabolic and pathological contexts. MCD feeding rapidly induces hepatic steatosis, inflammation, and fibrosis but generally causes weight loss and lacks several systemic metabolic abnormalities characteristic of human MAFLD (46–48). In contrast, high-fat and high-sugar dietary models more closely reproduce obesity, insulin resistance, dyslipidemia, and the gradual progression of human metabolic fatty liver disease (42, 49). Therefore, the MCD immunohistochemical results provide cross-model support for reduced hepatic FABP5 but cannot replace direct protein measurement in the original HFHF model. Further studies using newly generated or appropriately archived HFHF liver samples, quantitative immunoblotting, cell-specific co-immunostaining, and subcellular fractionation are needed to define the abundance and cellular distribution of hepatic FABP5.

PA treatment reduced *FABP5* and *PPAR-α* expression while increasing *CPT1* and *ACOX1* expression under some experimental conditions. PPARα is a ligand-activated nuclear receptor that regulates genes involved in fatty acid transport and mitochondrial and peroxisomal β-oxidation (50–52). CPT1 controls the entry of long-chain fatty acids into mitochondria and is inhibited by malonyl-CoA (53–55), whereas ACOX1 catalyzes the initial step of peroxisomal fatty acid β-oxidation (51). The increases in *CPT1* and *ACOX1* expression observed after PA treatment may therefore represent compensatory transcriptional responses to lipid overload rather than direct evidence of increased fatty acid oxidation. Importantly, malonyl-CoA-mediated inhibition applies primarily to CPT1-dependent mitochondrial fatty acid transport and should not be used to explain ACOX1 regulation. Moreover, changes in gene expression alone do not establish corresponding changes in enzyme activity or metabolic flux.

FABP5 overexpression increased *PPAR-α*, *CPT1*, and *ACOX1* mRNA expression and attenuated PA-induced intracellular lipid accumulation. These findings suggest that restoration of FABP5 may be associated with enhancement of a PPARα-related fatty acid oxidative program, thereby redirecting fatty acids away from triglyceride storage. A recent study also reported that an asprosin–FABP5 interaction modulated mitochondrial fatty acid oxidation through PPARα in an experimental MASLD context (26). Nevertheless, the present data do not establish PPARα as a necessary mediator of the lipid-lowering effect of FABP5. FABP5 is well established as a lipid chaperone capable of delivering ligands to PPARβ/δ, and the receptor activated by FABP5 may depend on ligand identity and cellular context (56). Furthermore, increased *PPAR-α* mRNA expression does not necessarily indicate increased PPARα protein abundance, ligand binding, or transcriptional activity. Experiments involving PPARα knockdown or pharmacological inhibition, followed by rescue approaches and PPAR-responsive reporter or target-gene assays, are required to determine whether PPARα is necessary for the observed FABP5 effect. Parallel evaluation of PPARβ/δ would also help distinguish which nuclear-receptor pathway mediates the response.

Several additional limitations should be acknowledged. First, both the animal transcriptomic cohort and the human serum cohort were relatively small. The human findings require replication in larger independent cohorts with adjustment for age, sex, adiposity, diabetes, dyslipidemia, medication use, and liver disease severity. Second, hepatic FABP5 protein abundance was not directly measured in the original HFHF-fed mice. Third, the *in vitro* experiments relied primarily on FABP5 overexpression in Huh-7 cells, which may not reproduce physiological FABP5 expression or primary hepatocyte biology. Complementary loss-of-function and dose-controlled rescue experiments are therefore needed. Fourth, only the mRNA expression of PPARα and selected fatty acid oxidation-related genes was measured. Protein abundance, nuclear-receptor activity, mitochondrial respiration, palmitate oxidation, acylcarnitine profiles, and peroxisomal β-oxidation were not determined. Measurement of oxygen consumption and fatty acid oxidation using extracellular-flux analysis would provide more direct evidence of mitochondrial metabolic function (57–60). Finally, the present study did not determine whether modulation of FABP5 improves hepatic steatosis or systemic metabolic dysfunction *in vivo*.

In conclusion, our results demonstrate a reproducible reduction in hepatic *Fabp5* expression in HFHF-fed mice and show that FABP5 overexpression attenuates PA-induced hepatocellular lipid accumulation while increasing the expression of fatty acid oxidation-related genes. Independent transcriptomic and proteomic datasets provide supportive, although not definitive, evidence for reduced hepatic FABP5 abundance in diet-induced fatty liver models. These findings warrant further investigation into the cell-specific and disease-stage-dependent roles of FABP5. At present, FABP5 should be regarded as a candidate regulator of hepatic lipid metabolism and an exploratory circulating marker rather than an established diagnostic biomarker or therapeutic target for MAFLD.

## Conclusion

Using ONT-based hepatic transcriptomic profiling, we identified 12 differentially expressed genes involved in lipid transport and metabolism in HFHF-fed mice, with all expression changes independently confirmed by RT-qPCR. Among these genes, *Fabp5* was consistently downregulated in the experimental model, while FABP5 overexpression attenuated PA-induced lipid accumulation in Huh-7 cells and was associated with increased expression of fatty acid oxidation-related genes. These findings identify FABP5 as a candidate regulator of hepatocellular lipid metabolism. Further cell-specific mechanistic studies and *in vivo* intervention experiments are required to determine its diagnostic and therapeutic relevance in MAFLD.

## Data Availability

The two retained raw Oxford Nanopore FASTQ files (WTchow and WT-HH) have been deposited in the NCBI Sequence Read Archive under BioProject accession PRJNA1514860. Processed transcriptomic data for all eight samples, including expression matrices, differential-expression results, quality-control statistics, transcript sequences, annotations, and metadata, are available in Figshare at https://doi.org/10.6084/m9.figshare.33292728. Raw FASTQ and signal files for the other six libraries were permanently purged after the commercial sequencing provider's retention period and cannot be recovered or regenerated, as confirmed by the provider. No raw data were reconstructed from processed results. Other supporting data are included in the article and its **Supplementary Material**.
